# Area-level socioeconomic status and variation in medication use among Medicaid enrollees with incident systemic lupus erythematosus, 2000 to 2004

**DOI:** 10.1186/ar3941

**Published:** 2012-09-27

**Authors:** CH Feldman, LT Hiraki, J Lui, GS Alarcón, MA Fischer, J Yazdany, KH Costenbader

**Affiliations:** 1 Brigham and Women's Hospital, Boston, MA, USA; 2 University of Alabama, Birmingham, AL, USA; 3 University of California, San Francisco, CA, USA

## Background

Differences in access to medications may be related to socioeconomic disparities in outcomes among individuals with systemic lupus erythematosus (SLE). We investigated county-level socioeconomic status (SES) and corticosteroid, hydroxychloroquine and immunosuppressant use among individuals in the low-income US Medicaid population.

## Methods

From the US Medicaid Analytic eXtract (MAX) data, containing all billing claims from 2000 to 2004, we identified adults, aged 18 to 65, with incident SLE (≥3 SLE ICD-9 codes (710.0), >30 days apart, with 24 months of prior enrollment without SLE claims). Sex, age, race/ethnicity, ZIP code, and geographic region were obtained. We determined health professional shortage areas, number of state-level rheumatologists, and county-level SES using a validated composite of US Census variables. Our outcome measures were use at any time of corticosteroids, hydroxychloroquine, or an immunosuppressant (azathioprine, mycophenolate mofetil, cyclophosphamide, cyclosporine or tacrolimus). We used multivariable logistic regression to examine relationships between sociodemographic variables and medication prescriptions.

## Results

Of 3,965 Medicaid enrollees with incident SLE, the mean age was 39.5 ± 12; 94.1% were female, 41.2% African American, 33.7% White, and 15.2% Hispanic. A total of 67.4% received corticosteroids, 49.5% received hydroxychloroquine, and 17.5% received an immunosuppressant. Adjusting for months of enrollment, 77.5% of patients in the lowest SES quartile received corticosteroids compared with 65.9% in the highest (OR = 1.8, 95% CI = 1.27 to 2.58). In our fully adjusted model (Table 1), increasing county-level SES among Medicaid enrollees with incident SLE was associated with a lower odds of receiving a corticosteroid prescription, but a higher odds of receiving an immunosuppressant prescription. Hydroxychloroquine use did not vary significantly by SES.

**Table 1:** Medication prescriptions among Medicaid enrollees with incident SLE, 2000 to 2004 according to county-level SES^a^

	Corticosteroids		Hydroxychloroquine		Immunosuppressants^b^	
	
	OR	95% CI	OR	95% CI	OR	95% CI
Increasing county-level SES	0.94	0.89-0.99	1.04	0.99-1.09	1.12	1.05-1.20

**^a^**Multivariable logistic regression models adjusted for number of Medicaid-enrolled months, age at first SLE claim, race/ethnicity, sex, geographic region, Health Professional Shortage Area, and state-level number of rheumatologists. ^b^Immunosuppressants include: azathioprine, mycophenolate mofetil, cyclophosphamide (oral and intravenous), cyclosporine or tacrolimus

## Conclusion

In higher SES areas, corticosteroid use among patients with incident SLE appears to be less frequent, while immunosuppressant use may be more frequent. Further studies are necessary to determine whether medication differences contribute to disparities in outcomes.

